# Supplementary material for: IconicITA: Iconicity ratings of the Italian affective lexicon
Source: PLoS One. 2025 Dec 8;20(12):e0337947. doi: 10.1371/journal.pone.0337947 (PMC12685184; doi:10.1371/journal.pone.0337947)
Supplement: Attachment — Submitted filename: R1_letter_response_to_reviewers.docx [file pone.0337947.s002.docx]

**PONE-D-25-25201**

**“IconicITA: Iconicity ratings of the Italian affective lexicon.”**

*PLOS One*

Bologna, 13^th^ October 2025

Dear Editor,

We would like to sincerely thank the reviewers for their careful reading of our manuscript and for their insightful and constructive comments. Their suggestions have been extremely valuable in helping us improve the clarity and quality of the paper. Below, we provide a detailed point-by-point response to reviewers’ comments.

We are grateful for the reviewers' insightful feedback and look forward to your reevaluation. We would like to thank the Editor for granting us the extensions for the submission.

We look forward to hearing from you at your earliest convenience.

Sincerely yours,

Tommaso Lamarra

*Corresponding author*

On behalf of the co-authors.

**Reply to reviewers’ comments**

We would like to sincerely thank the reviewers for their careful reading of our manuscript and for their insightful and constructive comments. Their suggestions have been extremely valuable in helping us improve the clarity and quality of the paper. Below, we provide a detailed point-by-point response to each of the issues raised.

**Reviewer #1:**

This ms presents iconicity ratings for 1121 Italian words, collected from two populations: L1 and L2 speakers. The number of words rated is quite low compared to other recent data sets (e.g. 13.000 for English in Winter et al. 2022), but the design does allow some interesting inferences and analyses, particularly because of the L1/L2 comparison. There is room for improvement particularly in the documentation of design decisions, scholarly embedding and in the description and interpretation of the L1/L2 comparison. I think the study requires revisions along the lines indicated below.

Methodology, documentation, analysis. It is good to have open data, but some aspects of the study are not yet documented in sufficient detail and others raise questions.

**1.** line 266 "Instructions were presented in Italian and in English". What were the instructions exactly? The supporting information that I could see included only an ethical approval form. We would need to see the full instructions as they were given, along with translations.

**Author’s response:** The instructions are stored in the online repository with all the materials related to the studies.

**2.** Line 268 "they had the option to select a "I do not know this word" button". The paper does not analyse these responses as far as I could see. Raters supplied on average 290 ratings (the mode is 234, some have double or quadruple that). Some have >90 "NA" values (presumably clicking "I do not know this word" a lot). There is sufficient variance among those NA rating numbers to wonder whether there is a relation to proficiency (presumably, less proficient speakers would more often not know some words). Matters like this need to be discussed and analysed.

**Author’s response:** We thank the reviewer for the comment. The intuition was indeed correct: in our L2 sample, we did encounter a significant (although weak) negative correlation between proficiency (as assessed with the LexITA score) and the number of “NA” values (i.e., words whose meaning was unknown to participants; r = -0.31, p = 0.009). We are not surprised by this trend: less proficient speakers are expected to know less words than more proficient speakers, especially since the proficiency test (LexITA) is highly focused on lexical knowledge. We now mention this negative correlation in § *Procedure*.

**3.** Exclusion criteria and their effects. In an effort to filter out bad raters, a comparison was done between a set of words presumed to be highly iconic versus the rest; people who did not show a large enough difference between the two were excluded. But the disproportionately skewed effect of applying this criterion should raise alarm bells: only 2 L1 speakers were excluded (3% of participants) but as many as 26 L2 speakers (31%) (line 261). This is a finding in and of itself, unless for some reason the assumption is that L2 speakers are more likely to be "bad raters" (but no such argument is made). What this shows is that a significant portion of L2 speakers of Italian (of what proficiency?) have trouble distinguishing sets of words that according to L1 speakers should be highly distinct in terms of iconicity. That speaks directly to the goal of the study so the exclusion here seems not a great idea. At the very least more analysis of this very skew is required.

**Author’s response:** We thank the reviewer for raising this important point. In the original submission, we excluded participants based on a t-test comparing ratings of highly iconic (onomatopoeic and phonosymbolic) words to the rest of the lexicon. As the reviewer noted, this procedure disproportionately affected L2 speakers, and another undesirable property is that it relied on a pre-defined subset of items assumed to be highly iconic based on the authors’ intuitions.
In the revision, we have replaced this criterion with a leave-one-out (LOO) reliability measure. For each participant, we correlated their ratings with the mean ratings of all other participants for the words they had in common. Participants were retained only if this correlation was positive and statistically significant (p < .05). This approach quantifies the reliability of each participant’s ratings relative to the group, and it avoids any assumptions about which items “should” be iconic.
Using this criterion, the number of retained participants is coherent across groups: out of 59 L1 speakers, 54 passed (91.53%); out of 69 L2 speakers, 57 passed (82.61%). While slightly more L2 participants were excluded, the difference is much smaller than with the original procedure, and the resulting samples are balanced and comparable.

Importantly, all of the main results replicate under this new exclusion scheme. The pattern of effects is in the same direction as in the original submission, and overall reliability and proficiency associations remain strong. Two differences are worth noting:

- The effect of Age of Acquisition (AoA) is now significant (β = –0.0348, SE = 0.0166, t = –2.1020, *p* = 0.0358), consistent with previous trends.
- The association between proficiency and correlation with English ratings, while positive, is no longer significant (*r* = 0.15, *p* = 0.261). This effect was already weaker than the corresponding association with Italian L1 ratings in the original analysis.

All other findings remain unchanged. Finally, we also corrected a reporting error in the *Participants* section. In the previous version, the participant counts were based on the number of annotation sessions rather than the number of unique annotators. We have now revised these numbers to accurately reflect unique participants.

**4.** Questions about proficiency. Proficiency does not figure in the abstract and is not prominent in the theoretical background; it first appears as a subquestion in RQ2. But proficiency *is* the subject of one of the only 2 figures. More critical discussion of this is needed throughout. What is Figure 2 supposed to show exactly, and why? Would one assume that correlation would have the same direction in both cases? Essentially this suggests that the higher a person's proficiency in Italian, the higher there iconicity ratings of Italian words correlate with L1 English speakers' ratings of English words — why would that be the case? I found this confusing and it was not addressed in the text or caption relating to the figure.

**Author’s response:** Our core prediction is that if iconicity ratings reflect form-meaning mappings in a given language (Italian), then greater experience with Italian should increase alignment with Italian L1 ratings. Alignment with English L1 ratings (same meanings but different forms) is included as a contrast condition, not a primary target: we do not expect a comparable proficiency effect there.
In the revised analyses, the correlation between Italian proficiency and alignment with Italian L1 ratings remains significant and positive (*r* = 0.34, *p* = .001). The corresponding correlation with English L1 ratings is smaller and non-significant, though still numerically positive. This asymmetry is exactly the pattern predicted by our construct-validity argument: as learners gain experience with Italian phonology and form-meaning mappings, their ratings converge toward Italian native judgments more than toward English native judgments. This shows that ratings genuinely capture language-specific form-meaning relationships rather than semantic information alone. We have clarified in the text and caption that the English panel is a comparison baseline to demonstrate selectivity (Italian > English), not an effect we expect to be equally strong.
Note that in the original version, we reported a significant proficiency-English correlation. With the updated preprocessing (new rater inclusion and imputation choices prompted by the reviewer and described in § Results), that relation remains in the same direction but is no longer significant. The Italian effect (the one that bears directly on our hypothesis) remains robust. We now state this explicitly in the *Results* and *Discussion* to avoid any ambiguity.

**5.** Scholarly embedding I. Iconicity ratings were first introduced for signed languages, in particular American Sign Language (Griffith et al. 1981) and British Sign Language (Vinson et al. 2008). The earliest work by Griffiths et al. is particularly important to engage with because it also collected ratings for L1 and L2 users of the language, and so is directly comparable to the current ms. Much of this literature is reviewed in Ortega 2017.

Scholarly embedding II. Though as the study notes L1/L2 comparisons are relatively rare, this makes the few prior studies also looking into L2 iconicity ratings this all the more important to engage with. I am aware of two recent ones for a range of spoken languages. McLean et al. 2022 (uncited in the ms) collect ratings for Japanese words from English L2 learners of Japanese and compare them to the Japanese L1 ratings collected by Thompson et al. 2020 (cited). And Punselie et al. (2024) collect L2 iconicity ratings in 5 languages and report strong correlations between (a) L2 iconicity ratings, (b) experimental guessability, and (c) a direct measure of cumulative form-meaning associations. These lines of work supply complementary evidence that also helps to test and establish the construct validity of iconicity so it would be a good idea to cover them in the theoretical background.

**Author’s response:** We thank the reviewer for this important comment. We have included several references to sign languages literature in the introduction. We also mentioned the suggested literature on spoken languages. However, we think there is an important difference between our L2 participants, who knew Italian at least at a B2 level, and, e.g., McLean et al 2022 participants, who had no knowledge of Japanese. We called this second type of participants naïve users, whereas we referenced as L2 speakers only participants who know the language to be rated, but have a different native/dominant language.

**6.** Questionable assumption. The ms proposes that "correlations between Italian L2 ratings and both Italian and English L1 ratings should be comparable, as the meaning of a word remains constant across languages" (line 204-5). No evidence is cited for that bold claim about the stability of meaning; and in fact, there is a lot of work showing that meanings can be warped by language-specific semantic spaces (e.g., Werner 1993; Majid et al. 2007; Blasi et al. 2023). On p. 9 it becomes clear this comparison relies on the translation-based alignment provided in the Italian ANEW materials. It would be good to clarify in abstract (line 42) and research questions (line 204ff) and to choose a more cautious wording that avoids the questionable assumption that meanings are "constant across languages".

**Author’s response:** We thank the reviewer for pointing this out. We made it clear both in the *Abstract* and on line 229 that we are considered lexical items in Italian and their translations into English.

**7.** Interpreting the L1/L2 comparison. The main emphasis in RQ and abstract is on the L2 Italian to L1 English comparison. As the ms says, "Critically, this correlation substantially exceeded the one observed between [etc.]" (line 381). But the Figure in this section focuses on proficiency. Only the grey line in panel 1 shows a relation to the baseline L1 ratings. Somehow the rhetorics of the ms made me expect more information and a deeper analysis of how exactly the ratings differed across these two. E.g., one might expect an analysis of points of divergence and convergence. Which items were most dissimilar? which were most similar? and how might this relate to semantic domains, sensory modality, linguistic form, word class or other possibly relevant features? (Punselie et al. 2024 do something like this in their study of L2 ratings in relation to a bunch of other features. They find that for words with meanings in the domain with sound, there are fewer discrepancies across iconicity measures.) This is the kind of analysis that is uniquely possible in a study like this, and the ms delivered less of it than expected.

**Author’s response:** We thank the reviewer for this important point. We fully agree that our initial submission emphasized the overall correlation patterns but did not sufficiently unpack *how* L1 and L2 judgments diverged at the item level. Following the reviewer’s suggestion, we have added new analyses, both exploratory and confirmatory, and results that directly address this. Specifically, we now provide a quadrant-based visualization of the L1–L2 standardized scores, in which each word is classified as high–high (HH), low–low (LL), high–low (HL), or low–high (LH). We highlight the ten most extreme items per quadrant (new Table 2 and new Figure 3). This makes clear which words are judged similarly across groups and which diverge most strongly. In addition, we have conducted confirmatory analyses to explain these divergences. We show that L2 judgments depart from L1 ratings in systematic ways: words that are phonologically similar to their English translations and words that are more frequent in Italian receive higher ratings in L2 than predicted by L1 ratings, whereas metaphorical or semantically opaque words show the opposite pattern. These results are now reported in the Results section and discussed in detail in the Discussion. Together, these additions provide the deeper analysis the reviewer requested: we identify specific items of convergence and divergence, and we interpret these differences in terms of psycholinguistic mechanisms (cross-linguistic transfer and learning by exposure). This strengthens the manuscript’s contribution by showing not only that L1 and L2 ratings correlate, but also *why* and *where* they diverge.

**8.** Copy-editing note: the numerical referencing style means that formulations that foreground the reference do not really work well. E.g., line 66 "Notably, [1] clarified that arbitrariness, systematicity and iconicity all contribute to language development and communication." This works if the authors are spelled out as in Chicago style, but for a numerical style it is better to move the reference to the end of the statement and lead with the finding: "Arbitrariness, systematicity and iconicity (...) [1]."

**Author’s response:** The manuscript was modified following this important point.

**9.** Nitpicking: line 121 "iconic words are the earliest words learned" — this is factually wrong. What the cited work and other work on AoA shows is that iconic words are relatively overrepresented among early words, meaning there are more of them than you might naively expect if words were encountered, used, and learned perfectly randomly. But there are still plenty of early words that are not all that iconic.

**Author’s response:** We corrected the sentence as suggested.

References cited

Blasi, Damián E., Joseph Henrich, Evangelia Adamou, David Kemmerer, and Asifa Majid. 2022. ‘Over-Reliance on English Hinders Cognitive Science’. Trends in Cognitive Sciences, October. doi:10.1016/j.tics.2022.09.015.

Griffith, Penny L., Jacques H. Robinson, and John M. Panagos. 1981. ‘Perception of Iconicity in American Sign Language by Hearing and Deaf Subjects’. Journal of Speech and Hearing Disorders 46 (4): 388–97. doi:10.1044/jshd.4604.388.

Majid, Asifa, Marianne Gullberg, Miriam van Staden, and Melissa Bowerman. 2007. ‘How Similar Are Semantic Categories in Closely Related Languages? A Comparison of Cutting and Breaking in Four Germanic Languages’. Cognitive Linguistics 18 (2): 179–94. doi:10.1515/COG.2007.007.

McLean, Bonnie, Michael Dunn, and Mark Dingemanse. 2023. ‘Two Measures Are Better than One: Combining Iconicity Ratings and Guessing Experiments for a More Nuanced Picture of Iconicity in the Lexicon’. Language and Cognition 5 (4). Cambridge University Press: 719–39. doi:10.1017/langcog.2023.9.

Ortega, Gerardo. 2017. ‘Iconicity and Sign Lexical Acquisition: A Review’. Frontiers in Psychology 8. doi:10.3389/fpsyg.2017.01280.

Punselie, Stella, Bonnie McLean, and Mark Dingemanse. 2024. ‘The Anatomy of Iconicity: Cumulative Structural Analogies Underlie Objective and Subjective Measures of Iconicity’. Open Mind 8 (September): 1191–1212. doi:10.1162/opmi_a_00162.

Thompson, Arthur Lewis, Kimi Akita, and Youngah Do. 2020. ‘Iconicity Ratings across the Japanese Lexicon: A Comparative Study with English’. Linguistics Vanguard 1 (ahead-of-print). De Gruyter Mouton. doi:10.1515/lingvan-2019-0088.

Vinson, David, Kearsy Cormier, Tanya Denmark, Adam Schembri, and Gabriella Vigliocco. 2008. ‘The British Sign Language (BSL) Norms for Age of Acquisition, Familiarity, and Iconicity’. Behavior Research Methods 40 (4): 1079–87. doi:10.3758/BRM.40.4.1079.

Werner, Oswald. 1993. ‘Short Take 10: Semantic Accent and Folk Definitions’. Cultural Anthropology Methods 5 (2): 6–7. doi:10.1177/1525822X9300500204.

**Reviewer #2:**

In their manuscript, “IconicITA: Iconicity ratings of the Italian affective lexicon,” the authors quantify iconicity in Italian affective words using ratings made by L1 and L2 Italian speakers. Replicating findings in other languages such as English, they find that the ratings of L1 Italian speakers are positively associated with perceptual strength in the haptic and auditory domain and negatively associated with frequency and concreteness. As ratings data were previously not available in Italian, this manuscript offers and important resource to the field and the data are fully available to other researchers. Furthermore, the authors also address a novel and interesting question about whether L2 speakers’ ratings should correspond more with L1 speakers’ or with the ratings of words in their native language. Here they find that L2 ratings are more correlated with L1 ratings, a finding with implications for how people perceive and consider iconicity. Thus, I believe this manuscript will be of interest to the broad readership of PLoS ONE. I have a few suggestions aimed at improving the manuscript further.

**1.** Theoretical background section does a good job of citing the relevant extant literature. However, it would help the reader consider why the various patterns exist in English as described in this literature and are expected to exist in Italian, if the authors were to go into a bit more detail about the mechanisms at play here. E.g., why are auditory and haptic words more iconic, and why is there a negative relationship with concreteness if both concreteness and iconicity are associated with word learning, etc?

**Author’s response:** We thank the reviewer for this comment. We discussed in more detail the relationship between these sensory modalities and iconicity ratings (see section *Iconicity ratings for Italian by L1 speakers*). In particular, we added a reference to a very recent manuscript (Winter and Strik Lievers, 2025) in which the authors report a dominant relationship between touch and auditory modalities through an extensive meta-analysis. As for the relationship with word learning, we have now specified better in the introduction that iconic words are highly common in the earliest acquired lexicon. This explains the negative correlation between AoA ratings and iconicity ratings (we specified this point more explicitly also in the discussion). The finding concerning concreteness is indeed puzzling, and we have reported it as such. We would like to stress, however, that concreteness ratings are a particularly complex metric: recent research has shown that they tend to conflate different types of semantic information, rather than capturing a single, homogeneous dimension (Troche et al., 2017; Bolognesi et al., 2020). In other words, what is summarized in a “concreteness” score may reflect a mix of perceptual, functional, and even contextual associations, which complicates its relationship with iconicity. This could partly account for the unexpected negative correlation between concreteness and iconicity ratings.

**2.** Can the authors comment on the connection between word learning literature in children learning an L1 and finding from L2 learners in the study. Are their implications for teaching a second language, e.g., targeting more highly iconic words?

**Author’s response:** We thank the reviewer for pointing out this nice link. We added a paragraph to the *Discussion*.

6. PLOS authors have the option to publish the peer review history of their article ([what does this mean?](https://track.editorialmanager.com/CL0/https:%2F%2Fjournals.plos.org%2Fplosone%2Fs%2Feditorial-and-peer-review-process%23loc-peer-review-history/1/010f01982af5509c-6abfac66-aef0-4b88-ad1f-2f63ce17d4c4-000000/iDN4Gx0-WcPlRUOQwUeezRXAIQa5RV0bUCmo9MjGX7k=220)). If published, this will include your full peer review and any attached files.


If you choose “no”, your identity will remain anonymous but your review may still be made public.


**Do you want your identity to be public for this peer review?** For information about this choice, including consent withdrawal, please see our [Privacy Policy](https://track.editorialmanager.com/CL0/https:%2F%2Fwww.plos.org%2Fprivacy-policy/1/010f01982af5509c-6abfac66-aef0-4b88-ad1f-2f63ce17d4c4-000000/BPOg0wChkT51hwsbPap2eF8ebAjYdgf1onxioZCjpKg=220).

Reviewer #1: **Yes:** M. Dingemanse

Reviewer #2: No
